# Infective Endocarditis From Pseudomonas aeruginosa and Group C Streptococcus

**DOI:** 10.7759/cureus.30904

**Published:** 2022-10-31

**Authors:** Abhinandan R Chittal, Shiavax J Rao, Pallavi Lakra, Rebecca Vietri, Hitesh Chawla

**Affiliations:** 1 Internal Medicine, MedStar Union Memorial Hospital, Baltimore, USA; 2 Nursing, MedStar Union Memorial Hospital, Baltimore, USA; 3 Cardiology, MedStar Heart and Vascular Institute, Baltimore, USA

**Keywords:** embolic phenomena, intravenous drug abusers (ivda), infective endocarditis, group c streptococcus endocarditis, group c streptococcus, streptococcus, pseudomonas endocarditis, pseudomonas, endocarditis

## Abstract

Endocarditis is a condition that is usually caused by an infection or inflammation of the endocardium. The disease is commonly seen among intravenous drug abusers, patients with intravenous catheters, and those who undergo cardiovascular and invasive dental procedures. Multiple different pathogens can cause endocarditis (bacterial, fungal, and viral) depending upon the patient's risk factors, epidemiology, and bacteria that are prevalent/endemic to the region. We present the case of a woman who had a history of polysubstance abuse, having presented to the hospital on multiple occasions with bacteremia with a previous admission for endocarditis, who developed a multi-bacterial infection at this presentation involving *Pseudomonas aeruginosa* and group C *Streptococcus*, which affected both the right and left side of the heart. In this paper, we reviewed common presentations of endocarditis caused by either bacteria, as well as recommendations for medical or surgical management of the condition.

## Introduction

Endocarditis is the integration of three terms put together: "endo," which means inside, "card," which means heart, and the suffix "itis," which means inflammation, hence meaning that it is an inflammation of the inner part of the heart (endocardium). Endocarditis is generally caused by bloodstream bacterial infections with risk factors including intravenous drug abuse (IVDA), intravenous catheters, intravenous procedures, recent dental infections, and patients with native valve disease or surgically replaced valves. The use of infected needles, commonly seen in patients with IVDA, is an important risk factor for endocarditis [1]. Group C *Streptococcus* (GCS) represents normal human flora [2]. Zoonotic infections are commonly seen with GCS, especially linked to people commonly engaged in farm work [3]. *Pseudomonas* endocarditis has been associated with poor outcomes, as well as high mortality and morbidity rates [4]. We present a complex clinical case of a woman with GCS and *Pseudomonas aeruginosa* endocarditis, complicated by septic pulmonary emboli and a septic embolic stroke.

This article was previously presented as a meeting abstract at the 2022 Society of Critical Care Medicine Annual Meeting in January 2022.

## Case presentation

A 34-year-old woman with a history of polysubstance abuse (most recently, intravenous heroin and oral fentanyl) presented to the hospital with complaints of feeling lethargic and sick. She noticed a wound on the lateral aspect of her right lower extremity and mid-shin and endorsed that she had been injecting heroin into the wound site regularly. She had a recent admission with a similar presentation where she was diagnosed with methicillin-sensitive *Staphylococcus aureus *(MSSA) bacteremia resulting in tricuspid valve endocarditis. She was evaluated by cardiac surgery at the time. However, the patient had left the hospital against medical advice.

On this admission, on initial physical examination, there was no acute distress, and she was alert and oriented to time, place, and person. A cardiac exam revealed a regular rate and rhythm with a holosystolic murmur in the tricuspid area, with a positive Rivero-Carvallo sign (murmur accentuated with inspiration). A skin exam revealed a necrotic looking 10 cm × 10 cm wound on the right lower extremity along the mid-shin. The remainder of the physical examination was unremarkable.

Laboratory diagnostics revealed serum sodium of 123 mmol/L (reference range 136-145 mmol/L), serum potassium of 3.4 mmol/L (reference range 3.4-4.5 mmol/L), blood urea nitrogen (BUN) 76 mg/dL (reference range 9-23 mg/dL), serum creatinine of 8.36 mg/dL (reference range 0.5-0.8 mg/dL; increased from the patient's previous baseline of 0.6 mg/dL), leukocytosis of 15,600 cells/uL (reference range 4,000-10,800 cells/uL), and anemia with hemoglobin of 9.5 g/dL (reference range 11-14.5 g/dL). Blood cultures were drawn at the time of admission, which grew *Pseudomonas aeruginosa *and beta-hemolytic GCS. The full culture and sensitivity report is given in Table 1. Based on culture and sensitivity reports, she was started on antimicrobial pharmacotherapy with vancomycin and ceftriaxone. Her hospital course was also unfortunately complicated by acute kidney injury, requiring the initiation of dialysis during this admission. 

**Table 1:** Culture and sensitivity data based on blood cultures on initial presentation. MIC interp: minimum inhibitory concentration interpretation; MIC dilutn: minimum inhibitory concentration dilution; R: resistant; S: susceptible (standard dosing regimen); I: susceptible (increased exposure).

	Beta-hemolytic *Streptococci*, group C		Pseudomonas aeruginosa	
Drug	MIC interp	MIC dilutn	MIC interp	MIC dilutn
Aztreonam			S	8
Cefepime			S	8
Cefotaxime	S	≤0.0625		
Ciprofloxacin			R	2
Clindamycin	S	0.0625		
Erythromycin	R	4		
Gentamicin			S	≤2
Levofloxacin			I	2
Penicillin	S	≤0.03125		
Piperacillin/tazobactam			S	8/4
Tobramycin			S	≤2
Vancomycin	S	0.5		

**Figure 1:**
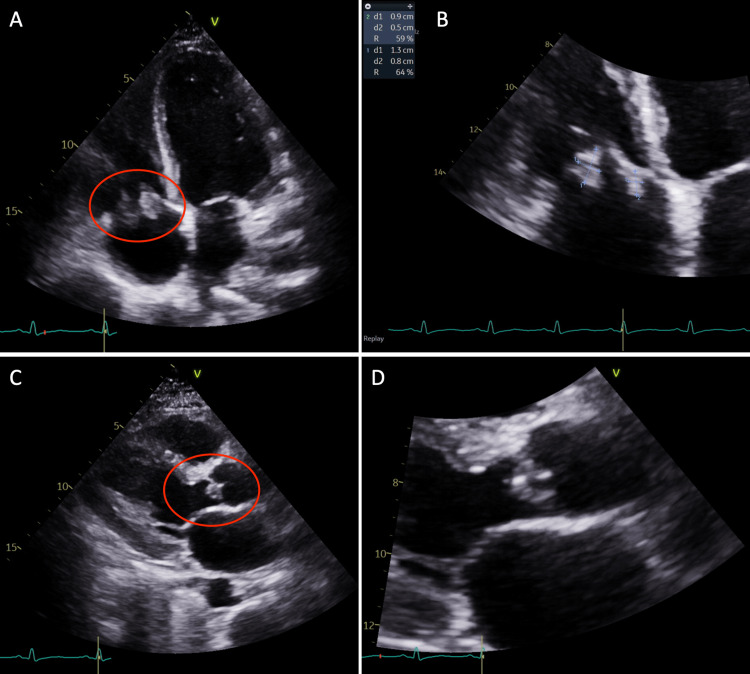
Transthoracic echocardiogram apical four-chamber view (A) and magnified view of the tricuspid valve (B) revealing echodensities measuring 0.8 cm × 1.3 cm and 0.9 cm × 0.5 cm on the atrial side of the anterior and septal tricuspid valve leaflets. The parasternal long-axis view (C) and magnified view of the aortic valve (D) revealing a <1 cm echodensity on the ventricular side of the noncoronary cusp of the aortic valve.

During the admission, the patient complained of persistent shortness of breath and, given her history of endocarditis in the past, a repeat echocardiogram was performed. The echocardiogram showed an ejection fraction of 55% to 60%, with echodensities measuring 0.8 cm × 1.3 cm and 0.9 cm × 0.5 cm on the atrial side of the anterior and septal tricuspid valve leaflets (Figure 1). There was also a <1 cm echodensity on the ventricular side of the noncoronary cusp of the aortic valve, which was concerning for endocarditis (Figure 1). There was mild aortic regurgitation present, with no evidence of pericardial effusion. The patient was re-evaluated by cardiothoracic surgery at this hospitalization for surgical/percutaneous intervention; however, the patient again left the hospital against medical advice, as she did not want to stay in the hospital and stated that her requirement for pain management was not being met.

**Figure 2:**
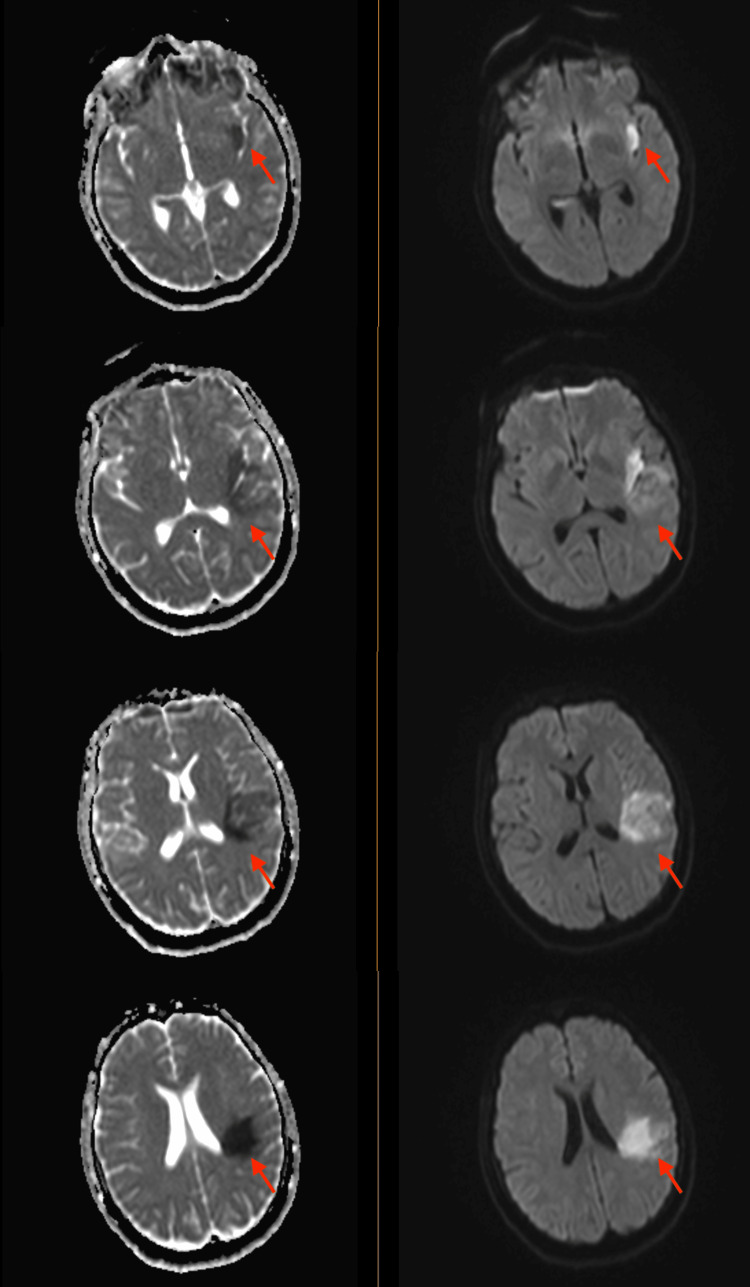
MRI of the brain showing early subacute ischemia in the left insula posteriorly and frontoparietal lobe in the middle cerebral artery territory, without any signs of hemorrhage, midline shift or cerebral edema.

**Figure 3:**
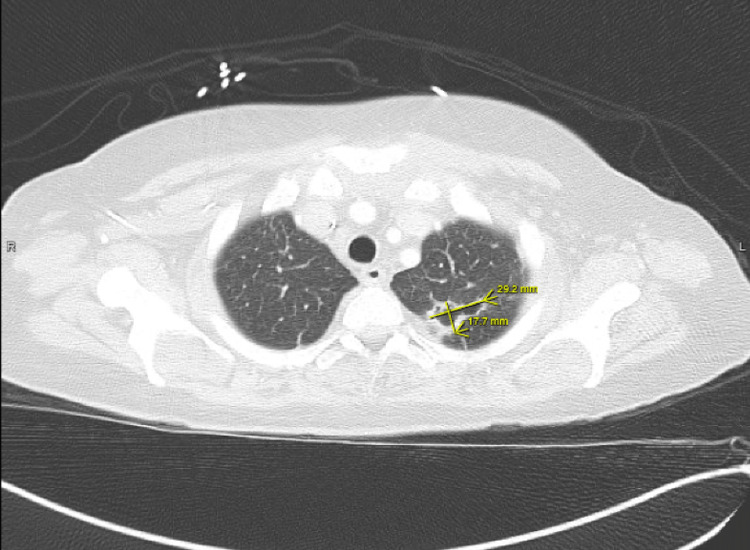
CT scan of the chest (axial slice, lung window) showing septic embolus to left lung field.

**Figure 4:**
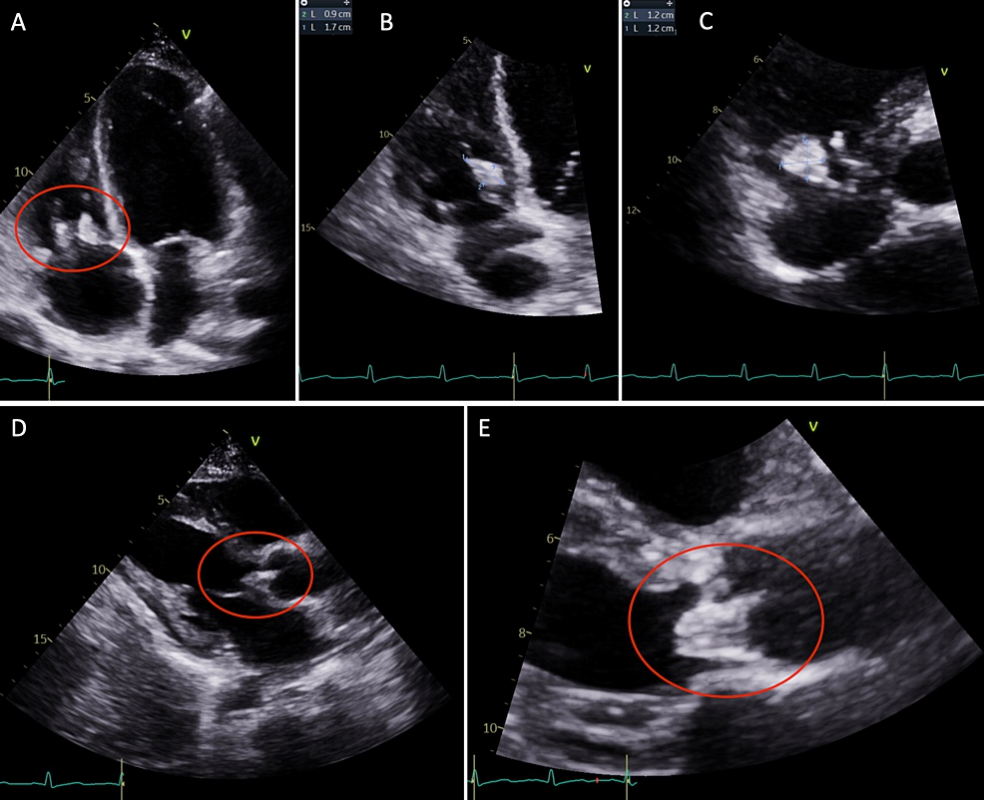
Repeat transthoracic echocardiogram revealing the increasing size of valvular vegetations. Apical four-chamber view (A) and magnified views of the tricuspid valve (B) and (C) showing a 1.2 cm × 1.2 cm echodensity attached to the anterior leaflet and a 0.9 cm × 1.7 cm echodensity attached to the septal leaflet. The parasternal long-axis view (D) and magnified view of the aortic valve (E) showing a 1.7 cm × 1.2 cm echodensity on the noncoronary cusp of the aortic valve.

A few weeks later, the patient returned to the hospital with right-sided hemiparesis (strength of 3/5 in all muscle groups of the right upper and lower extremity), retained power on the left side, and impaired fine muscle movements on the right hand without any involuntary movements. She also showed mild right-sided facial weakness. At this time, an MRI of the brain was pursued, which showed early subacute ischemia in the left insula posteriorly and the frontoparietal lobe in the middle cerebral artery territory, without any signs of hemorrhage, midline shift, or cerebral edema (Figure 2). The patient continued to have shortness of breath during this admission. CT angiography of the chest revealed multiple bilateral pulmonary nodules, with a left upper lobe nodule being centrally cavitary and a dominant nodule in the right upper lobe measuring 2.5 cm with a small internal focus of air. These findings were concerning for septic emboli (Figure 3). At this time, a repeat transthoracic echocardiogram revealed the increasing size of vegetation on both the tricuspid and aortic valves (Figure 4).

Antibiotics were switched from vancomycin to linezolid; however, in the setting of linezolid use, the patient developed anemia and thrombocytopenia with a hemoglobin nadir of 6.9 g/dL and a platelet nadir of 78,000 cells/uL (baseline platelet count of 177,000 cells/uL). Consequently, the antibiotic regimen was further modified to cefepime and daptomycin. On account of medication non-compliance and continued IVDA, she was deemed to be a non-ideal and high-risk candidate for surgical intervention at this time. She was continued on a six-week course of intravenous cefepime and daptomycin, with planned outpatient follow-up with cardiac surgery. Unfortunately, the patient was lost to follow-up and did not present to her outpatient appointments or back to the hospital since discharge.

## Discussion

Infective endocarditis is an infection of the endocardial surfaces of cardiac tissue and may include one or more valves, mural endocardium of the septum, or septal defect. It may lead to valvular insufficiency or congestive heart failure and can be complicated by myocardial or paravalvular abscesses [5]. Endocarditis can primarily be classified under three categories: native valve endocarditis (NVE), prosthetic valve endocarditis, and IVDA endocarditis [6]. Major risk factors for NVE include rheumatic valvular disease (30% of NVE), primarily involving the mitral valve. Congenital heart disease accounts for 15% of NVE with common etiologies including patent ductus arteriosus, tetralogy of Fallot, and ventricular septal defect, and these are observed to be lesions with surgical high flow. Mitral valve prolapse with an associated murmur accounts for about 20% of NVE; degenerative heart disease (e.g., aortic stenosis from bicuspid aortic valve), Marfan's and syphilitic heart disease are less common causes [6]. Prosthetic valve endocarditis is frequently associated with complications such as local abscess, fistula formation, and valve dehiscence. Infective endocarditis is the most sinister complication of IVDA, given that bacteria can be introduced into the bloodstream via contaminated needles. The incidence of infective endocarditis among intravenous drug abusers is between 2% and 5% per year and accounts for 5%-20% of hospital admissions, with 5%-10% of the overall mortality rate among that demographic [7].

Seventy percent of all infections are caused by *Streptococcus* species including *Streptococcus viridans, S. bovis,* and *Enterococci*. *Staphylococcus *species are responsible for 25% of NVE [6]. Prosthetic valve endocarditis is caused by a variety of different pathogens based on the onset of the disease. Early prosthetic valve endocarditis is most commonly caused by *Staphylococcus aureus *and *S. epidermidis. *These organisms can include methicillin-resistant *S. aureus* (MRSA). The late disease is commonly caused by *Streptococcus *species [6].

In terms of IVDA endocarditis, MSSA is the most common etiology. Other associated bacteria include *Streptococci, Enterococci, *gram-negative rods, and *Candida *species. In patients with IVDA who present with left-sided endocarditis, the mortality is between 20% and 30% and with surgery decreases only to 15%-25%. IVDA patients who present with gram-negative rods or fungal endocarditis have a worse prognosis with mortality being significantly higher [7].

GCS can cause infections, being a part of normal human flora as well as being commonly seen in horses. Infections are common among intravenous drug abusers. *Streptococcus dysgalactiae *subsp. equisimilis is the primary human pathogen [2]. Bacteremia is associated with 25%-59% of patients with GCS, with endocarditis occurring in up to 47% of these patients. High rates of embolic disease are noted with GCS endocarditis-up to 45% of cases develop the embolic disease, with a high mortality rate of up to 13% [8]. Treatment choices mainly include penicillin or ceftriaxone, recommended for four to six weeks, with the addition of gentamicin for the first two weeks. Early cardiac surgery may improve survival in these patients [9].

*Pseudomonas* endocarditis is rare and occurs in about 3% of the cases of endocarditis. It, however, is strongly associated with IVDA, or in patients with prosthetic valves and pacemakers [10]. More than 90% of cases of *Pseudomonas *endocarditis have been reported in patients with IVDA who had no prior structural heart disease. It tends to be in specific geographic locations, likely due to abusers of intravenous drugs in these areas frequently using pentazocine and tripelennamine, presumably mixing them with contaminated water [11]. Recommendations for antimicrobial therapy for *Pseudomonas *endocarditis include two intravenous antipseudomonal antibiotics from different classes based on isolate susceptibility. One of the antibiotics is usually an aminoglycoside, unless the use is precluded based on renal function. The duration of therapy should be for at least six weeks. There are no large-scale studies regarding combination versus monotherapy for *Pseudomonas* endocarditis given that it is an uncommon infection; however, the vast majority of published data describes combination antimicrobial therapy [11].

In terms of surgical criteria for infective endocarditis, most recommendations are based on observational studies that have been related to no benefit in patients who received surgical intervention versus medical therapy [12]. Surgical management of infective endocarditis is recommended in patients where there is an association between surgery and a reduced risk of mortality: patients who have complicated infective endocarditis including heart failure, intracardiac abscess or fistula formation, native valve staph aureus endocarditis or evidence of systemic embolization [13].

In patients with heart failure due to valvular lesions, especially regurgitation, there is a small window for surgical intervention [13]. There is some controversy regarding the timing of surgery post-embolic stroke; however, early studies showed deterioration of patients' clinical status after surgical intervention [13]. The 2015 American Heart Association (AHA) infective endocarditis guidelines contain recommendations that are similar to the 2017 AHA/American College of Cardiology focused update on valvular heart disease, which notes that it is reasonable to delay valve surgery for at least four weeks after a major ischemic stroke or intracranial hemorrhage, if the patient is hemodynamically stable [14].

Our patient did meet the criteria for surgical intervention. She had evidence of early-onset heart failure and embolic lesions to the lungs (Figure 3). However, the patient's compliance issues and continued drug use made her a less than ideal candidate to pursue surgical management at that time. She decided to leave against medical advice and subsequently presented with paresis and embolic stroke, further deterioration of renal function (requiring initiation of dialysis), and worsening respiratory status (with septic emboli), which further complicated the overall management.

## Conclusions

Infective endocarditis is a common complication of IVDA. GCS is a part of normal human flora and can cause endocarditis in intravenous drug abusers and individuals handling horses. *Pseudomonas* endocarditis is rare and is usually isolated to specific geographic areas as drug abusers may use contaminated water. *Pseudomonas* endocarditis has a poor prognosis with a mortality rate of between 25% and 40%. Patients with left-sided endocarditis typically have poorer outcomes with a high mortality rate. Early surgical intervention is beneficial in certain clinical situations such as persistent bacteremia, development of heart failure, infections with highly virulent organisms, prosthetic valve endocarditis and disease complicated by paravalvular abscess and embolic events.
